# 3,6,8-Tribromo­quinoline

**DOI:** 10.1107/S1600536810045484

**Published:** 2010-11-13

**Authors:** Ísmail Çelik, Mehmet Akkurt, Salih Ökten, Osman Çakmak, Santiago García-Granda

**Affiliations:** a Department of Physics, Faculty of Arts and Sciences, Cumhuriyet University, 58140 Sivas, Turkey; b Department of Physics, Faculty of Sciences, Erciyes University, 38039 Kayseri, Turkey; c Department of Chemistry, Faculty of Art and Science, ­Gaziosmanpaşa University, 60240 Tokat, Turkey; d Departamento Química Física y Analítica, Facultad de Química, Universidad Oviedo, C/ Julián Clavería, 8, 33006 Oviedo (Asturias), Spain

## Abstract

The title mol­ecule, C_9_H_4_Br_3_N, is almost planar, the maximum deviation being 0.110 (1) Å. The crystal structure is stabilized by weak aromatic π–π inter­actions [centroid–centroid distance = 3.802 (4) Å] between the pyridine and benzene rings of the quinoline ring systems of adjacent mol­ecules.

## Related literature

For background to the synthesis of natural biologically active quinoline derivatives and for the synthesis of the title compound, see: Şahin *et al.* (2008 ▶). For the structure of 6,8-dibromo­quinoline, see: Çelik *et al.* (2010 ▶).
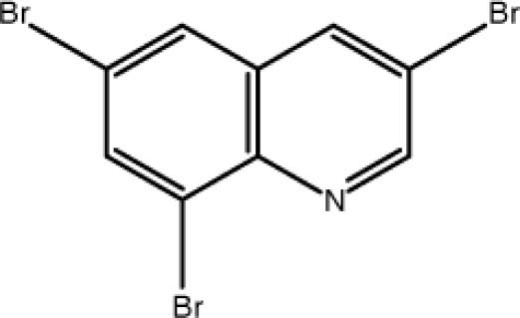


## Experimental

### 

#### Crystal data

C_9_H_4_Br_3_N
*M* *_r_* = 365.83
Monoclinic, 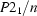

*a* = 3.9810 (2) Å
*b* = 12.4176 (4) Å
*c* = 19.7419 (6) Å
β = 92.827 (3)°
*V* = 974.74 (7) Å^3^
*Z* = 4
Cu *K*α radiation
μ = 14.93 mm^−1^
*T* = 296 K
0.51 × 0.06 × 0.03 mm

#### Data collection

Oxford Diffraction Xcalibur diffractometer with a Ruby Gemini CCD detector
Absorption correction: multi-scan (*CrysAlis PRO*; Oxford Diffraction, 2010 ▶) *T*_min_ = 0.049, *T*_max_ = 0.663
3688 measured reflections
1816 independent reflections
1484 reflections with *I* > 2σ(*I*)
*R*_int_ = 0.060

#### Refinement

*R*[*F* ^2^ > 2σ(*F* ^2^)] = 0.054
*wR*(*F* ^2^) = 0.150
*S* = 1.05
1816 reflections
118 parameters
H-atom parameters constrained
Δρ_max_ = 1.45 e Å^−3^
Δρ_min_ = −0.80 e Å^−3^

### 

Data collection: *CrysAlis PRO* (Oxford Diffraction, 2010 ▶); cell refinement: *CrysAlis PRO*; data reduction: *CrysAlis PRO*; program(s) used to solve structure: *SIR97* (Altomare *et al.*, 1999 ▶); program(s) used to refine structure: *SHELXL97* (Sheldrick, 2008 ▶); molecular graphics: *ORTEP-3 for Windows* (Farrugia, 1999 ▶); software used to prepare material for publication: *WinGX* (Farrugia, 1997 ▶) and *PLATON* (Spek, 2009 ▶).

## Supplementary Material

Crystal structure: contains datablocks global, I. DOI: 10.1107/S1600536810045484/pv2350sup1.cif

Structure factors: contains datablocks I. DOI: 10.1107/S1600536810045484/pv2350Isup2.hkl

Additional supplementary materials:  crystallographic information; 3D view; checkCIF report

## Figures and Tables

**Fig. 1.**
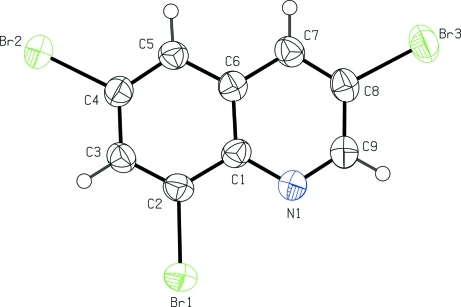
The title molecule with the atom numbering scheme. Displacement ellipsoids for have been drawn at the 50% probability level.

## supplementary crystallographic information

### Comment

The presence of quinoline skeleton in the framework of pharmacologically active compounds and natural products has spurred on the development of different strategies for their synthesis. The lithium–halogen exchange reaction of the title compound (I) may serve for the synthesis of natural biologically active quinoline derivatives, such as quinine, pentaquine, and plasmoquine (Şahin *et al.*, 2008). In this paper we report a one pot synthesis of (I) with high yield (90%) and its crystal structure.

The title molecule is almost planar, with the maximum and minimum deviations from the mean plane being 0.110 (1) and -0.001 (6) Å for Br2 and C4, respectively. Its crystal structure is stabilized by weak π–π stacking interactions between the pyridine and benzene rings of the quinoline ring systems of the adjacent molecules [*Cg*1···*Cg*2*^i^* = 3.802 (4) Å; symmetry code: (i) 1 + *x*, *y*, *z*; *Cg*1 and *Cg*2 are centroids of the N1/C1/C6–C9 pyridine and C1–C6 benzene rings of the quinoline ring system, respectively].

The crystal structure of 6,8-dibromoquinoline has been reported recently Çelik *et al.* (2010).

### Experimental

6,8-Dibromo-1,2,3,4-tetrahydroquinoline was synthesized according to the literature method (Şahin *et al.*, 2008). To a solution of 6,8-dibromo-1,2,3,4-tetrahydroquinoline (0.5 g, 3.75 mmol, 1 eq) in CHCl_3_ (20 ml) was dropped bromine (1.8 g, 11.25 mmol, 3 eq) in CHCl_3_ (10 ml) over 5 min in the dark and at room temperature. After completion of the reaction (bromine consumed completely, 3 days), the solid was dissolved in CHCl_3_ (35 ml) and the organic layer was washed with 5% NaHCO_3_ solution (3x20 ml) and dried over Na_2_SO_4_. After evaporation of the solvent, the crude material (1.32 g) was passed through a short alumina column eluting with EtOAc–hexane (1:12, 75 ml) (hexane/ethyl acetate, 9:1, *R_f_*= 0.65). Colourless solid residue was obtained. The mixture was recrystallized from the solvent (benzene) in a freezer (263 K) to give pure 3,6,8-tribromoquinoline in 90% yield (1.24 g) if the form of colourless neddle shaped crystals; m.p. 441–443 K.

### Refinement

H atoms were included in geometric positions with C—H = 0.93 Å and refined by using a riding model [*U*_iso_(H) = 1.2*U*_eq_(C)]. The highest peak in the final difference map was located 0.92Å from Br2, while the deepest hole was located 1.05Å from Br3.

### Crystal data

**Table:**

C_9_H_4_Br_3_N	*F*(000) = 680
*M**_r_* = 365.83	*D*_x_ = 2.493 Mg m^−^^3^
Monoclinic, *P*2_1_/*n*	Cu *K*α radiation, λ = 1.5418 Å
Hall symbol: -P 2yn	Cell parameters from 2025 reflections
*a* = 3.9810 (2) Å	θ = 3.6–70.4°
*b* = 12.4176 (4) Å	µ = 14.93 mm^−^^1^
*c* = 19.7419 (6) Å	*T* = 296 K
β = 92.827 (3)°	Needle, colourless
*V* = 974.74 (7) Å^3^	0.51 × 0.06 × 0.03 mm
*Z* = 4	

### Data collection

**Table:**

Oxford Diffraction Xcalibur diffractometer with a Ruby Gemini CCD detector	1816 independent reflections
Radiation source: Enhance (Cu) X-ray Source	1484 reflections with *I* > 2σ(*I*)
graphite	*R*_int_ = 0.060
Detector resolution: 10.2673 pixels mm^-1^	θ_max_ = 70.6°, θ_min_ = 5.7°
ω scans	*h* = −4→4
Absorption correction: multi-scan (*CrysAlis PRO*; Oxford Diffraction, 2010)	*k* = −9→15
*T*_min_ = 0.049, *T*_max_ = 0.663	*l* = −22→24
3688 measured reflections	

### Refinement

**Table:**

Refinement on *F*^2^	Primary atom site location: structure-invariant direct methods
Least-squares matrix: full	Secondary atom site location: difference Fourier map
*R*[*F*^2^ > 2σ(*F*^2^)] = 0.054	Hydrogen site location: inferred from neighbouring sites
*wR*(*F*^2^) = 0.150	H-atom parameters constrained
*S* = 1.05	*w* = 1/[σ^2^(*F*_o_^2^) + (0.1066*P*)^2^] where *P* = (*F*_o_^2^ + 2*F*_c_^2^)/3
1816 reflections	(Δ/σ)_max_ = 0.001
118 parameters	Δρ_max_ = 1.45 e Å^−^^3^
0 restraints	Δρ_min_ = −0.79 e Å^−^^3^

### Special details

**Table:**

Geometry. Bond distances, angles *etc*. have been calculated using the rounded fractional coordinates. All su's are estimated from the variances of the (full) variance-covariance matrix. The cell e.s.d.'s are taken into account in the estimation of distances, angles and torsion angles
Refinement. Refinement on *F*^2^ for ALL reflections except those flagged by the user for potential systematic errors. Weighted *R*-factors *wR* and all goodnesses of fit *S* are based on *F*^2^, conventional *R*-factors *R* are based on *F*, with *F* set to zero for negative *F*^2^. The observed criterion of *F*^2^ > σ(*F*^2^) is used only for calculating -*R*-factor-obs *etc*. and is not relevant to the choice of reflections for refinement. *R*-factors based on *F*^2^ are statistically about twice as large as those based on *F*, and *R*-factors based on ALL data will be even larger.

### Fractional atomic coordinates and isotropic or equivalent isotropic displacement parameters (Å^2^)

**Table:**

	*x*	*y*	*z*	*U*_iso_*/*U*_eq_	
Br1	0.6380 (2)	0.29099 (6)	0.90189 (4)	0.0568 (3)	
Br2	0.11453 (19)	−0.12590 (6)	0.92944 (4)	0.0525 (3)	
Br3	0.7497 (2)	0.08258 (7)	0.56672 (4)	0.0608 (3)	
N1	0.7203 (14)	0.2168 (5)	0.7553 (3)	0.0440 (17)	
C1	0.5666 (16)	0.1396 (5)	0.7921 (3)	0.0396 (17)	
C2	0.5134 (15)	0.1576 (5)	0.8619 (3)	0.0404 (17)	
C3	0.3758 (16)	0.0808 (5)	0.9010 (3)	0.0428 (17)	
C4	0.2822 (16)	−0.0184 (5)	0.8725 (3)	0.0425 (17)	
C5	0.3078 (15)	−0.0384 (5)	0.8042 (3)	0.0405 (17)	
C6	0.4583 (15)	0.0399 (5)	0.7636 (3)	0.0387 (17)	
C7	0.5126 (16)	0.0209 (5)	0.6946 (3)	0.0426 (17)	
C8	0.6665 (16)	0.0996 (5)	0.6597 (3)	0.0429 (17)	
C9	0.7713 (17)	0.1961 (6)	0.6919 (3)	0.0462 (17)	
H3	0.34400	0.09420	0.94660	0.0520*	
H5	0.22720	−0.10250	0.78520	0.0480*	
H7	0.44560	−0.04330	0.67380	0.0510*	
H9	0.88170	0.24730	0.66670	0.0550*	

### Atomic displacement parameters (Å^2^)

**Table:**

	*U*^11^	*U*^22^	*U*^33^	*U*^12^	*U*^13^	*U*^23^
Br1	0.0752 (6)	0.0456 (4)	0.0506 (5)	−0.0125 (3)	0.0119 (4)	−0.0120 (3)
Br2	0.0622 (5)	0.0498 (5)	0.0464 (4)	−0.0097 (3)	0.0107 (3)	0.0057 (3)
Br3	0.0798 (6)	0.0652 (5)	0.0385 (4)	0.0047 (4)	0.0132 (3)	−0.0003 (3)
N1	0.053 (3)	0.040 (3)	0.039 (3)	−0.003 (2)	0.003 (2)	0.002 (2)
C1	0.043 (3)	0.032 (3)	0.044 (3)	0.002 (2)	0.003 (2)	0.002 (2)
C2	0.044 (3)	0.037 (3)	0.040 (3)	0.002 (2)	0.000 (2)	−0.004 (2)
C3	0.042 (3)	0.050 (3)	0.037 (3)	−0.003 (3)	0.007 (2)	−0.006 (3)
C4	0.046 (3)	0.039 (3)	0.043 (3)	0.000 (3)	0.006 (2)	0.003 (2)
C5	0.045 (3)	0.038 (3)	0.038 (3)	0.000 (2)	−0.004 (2)	0.000 (2)
C6	0.039 (3)	0.038 (3)	0.039 (3)	0.006 (2)	0.000 (2)	0.003 (2)
C7	0.048 (3)	0.041 (3)	0.039 (3)	0.007 (3)	0.005 (2)	−0.003 (2)
C8	0.045 (3)	0.047 (3)	0.037 (3)	0.010 (3)	0.004 (2)	0.003 (2)
C9	0.053 (3)	0.047 (3)	0.039 (3)	−0.002 (3)	0.006 (3)	0.004 (2)

### Geometric parameters (Å, °)

**Table:**

Br1—C2	1.891 (6)	C4—C5	1.380 (8)
Br2—C4	1.888 (6)	C5—C6	1.412 (9)
Br3—C8	1.893 (6)	C6—C7	1.410 (8)
N1—C1	1.366 (9)	C7—C8	1.359 (9)
N1—C9	1.303 (8)	C8—C9	1.410 (9)
C1—C2	1.422 (8)	C3—H3	0.9300
C1—C6	1.418 (9)	C5—H5	0.9300
C2—C3	1.359 (9)	C7—H7	0.9300
C3—C4	1.397 (9)	C9—H9	0.9300
			
Br1···N1	3.070 (6)	C7···C8^ii^	3.542 (9)
Br1···Br3^i^	3.6969 (12)	C7···Br1^iv^	3.738 (6)
Br1···C7^i^	3.738 (6)	C8···C7^viii^	3.542 (9)
Br2···C4^ii^	3.696 (6)	C9···Br2^ix^	3.553 (7)
Br2···C9^iii^	3.553 (7)	C9···C6^viii^	3.587 (9)
Br3···Br1^iv^	3.6969 (12)	C5···H9^iv^	2.9800
Br3···Br3^v^	3.8186 (12)	H3···Br2^vii^	3.1500
Br1···H7^i^	3.0800	H3···Br2^vi^	3.2000
Br2···H9^iii^	3.1000	H5···H7	2.5100
Br2···H3^vi^	3.2000	H5···H9^iv^	2.5800
Br2···H9^iv^	3.2400	H7···H5	2.5100
Br2···H3^vii^	3.1500	H7···Br1^iv^	3.0800
N1···Br1	3.070 (6)	H9···Br2^ix^	3.1000
C4···Br2^viii^	3.696 (6)	H9···Br2^i^	3.2400
C5···C6^ii^	3.572 (8)	H9···C5^i^	2.9800
C6···C5^viii^	3.572 (8)	H9···H5^i^	2.5800
C6···C9^ii^	3.587 (9)		
			
C1—N1—C9	117.9 (6)	C5—C6—C7	121.5 (6)
N1—C1—C2	119.8 (6)	C6—C7—C8	117.7 (6)
N1—C1—C6	122.5 (6)	Br3—C8—C7	121.1 (5)
C2—C1—C6	117.7 (5)	Br3—C8—C9	118.0 (5)
Br1—C2—C1	119.6 (5)	C7—C8—C9	120.9 (6)
Br1—C2—C3	118.8 (5)	N1—C9—C8	123.0 (6)
C1—C2—C3	121.6 (6)	C2—C3—H3	120.00
C2—C3—C4	119.8 (6)	C4—C3—H3	120.00
Br2—C4—C3	118.6 (4)	C4—C5—H5	120.00
Br2—C4—C5	120.0 (5)	C6—C5—H5	121.00
C3—C4—C5	121.4 (6)	C6—C7—H7	121.00
C4—C5—C6	119.0 (6)	C8—C7—H7	121.00
C1—C6—C5	120.3 (5)	N1—C9—H9	119.00
C1—C6—C7	118.1 (5)	C8—C9—H9	118.00
			
C9—N1—C1—C2	178.4 (6)	C2—C3—C4—Br2	176.6 (5)
C9—N1—C1—C6	−0.9 (9)	C2—C3—C4—C5	−3.9 (10)
C1—N1—C9—C8	1.9 (10)	Br2—C4—C5—C6	−175.1 (5)
N1—C1—C2—Br1	2.9 (8)	C3—C4—C5—C6	5.4 (9)
N1—C1—C2—C3	−176.9 (6)	C4—C5—C6—C1	−3.0 (9)
C6—C1—C2—Br1	−177.8 (4)	C4—C5—C6—C7	175.5 (6)
C6—C1—C2—C3	2.5 (9)	C1—C6—C7—C8	0.1 (9)
N1—C1—C6—C5	178.5 (6)	C5—C6—C7—C8	−178.4 (6)
N1—C1—C6—C7	−0.1 (9)	C6—C7—C8—Br3	−179.7 (5)
C2—C1—C6—C5	−0.9 (9)	C6—C7—C8—C9	0.8 (9)
C2—C1—C6—C7	−179.5 (6)	Br3—C8—C9—N1	178.6 (5)
Br1—C2—C3—C4	−179.9 (5)	C7—C8—C9—N1	−1.9 (10)
C1—C2—C3—C4	−0.2 (9)		

Symmetry codes: (i) −*x*+3/2, *y*+1/2, −*z*+3/2; (ii) *x*−1, *y*, *z*; (iii) −*x*+1/2, *y*−1/2, −*z*+3/2; (iv) −*x*+3/2, *y*−1/2, −*z*+3/2; (v) −*x*+1, −*y*, −*z*+1; (vi) −*x*+1, −*y*, −*z*+2; (vii) −*x*, −*y*, −*z*+2; (viii) *x*+1, *y*, *z*; (ix) −*x*+1/2, *y*+1/2, −*z*+3/2.

### Table 1 π–π Stacking interactions in the title structure

Cg1 and Cg2 are centroids of the N1/C1/C6–C9 pyridine and C1–C6 benzene rings of the quinoline ring system, respectively.

**Table:**

Ring 1	Ring 2(sym)	(Ring 1)···(Ring 2) (Å)
Cg1	Cg2^i^	3.802 (4)

^i^: 1+x, y, z.
